# Risk of cardiac death in diabetic haemodialysis patients increased due to thyroid problems

**Published:** 2016

A prospective study found that diabetic haemodialysis patients’ sub-clinical hyperthyroidism and euthyroid sick syndrome may increase the risk of sudden cardiac-related deaths. Dr Christiane Drechsler, of University Hospital Würzburg in Würzburg, Germany, and colleagues conducted a study that included 1 000 patients undergoing haemodialysis for diabetes.

Of those patients, 78.1% had euthyroidism, 13.7% had sub-clinical hyperthyroidism, 1.6% had sub-clinical hypothyroidism and 5.4% had euthyroid sick syndrome. Patients with euthyroidism were compared with those who had sub-clinical hyperthyroidism and euthyroid sick syndrome with regard to which group showed an increased short-term (within a 12-month period) risk of sudden cardiac death.

It showed that patients who had euthyroidism had a 2.0-fold increased short-term risk of sudden cardiac death, and those who had sub-clinical hyperthyroidism and euthyroid sick syndrome had a 2.7-fold increase. The results showed that euthyroid sick syndrome was associated with a three-fold increased risk of short-term mortality, but in the long term (two to four years) it showed no increased risk.

The study revealed that sub-clinical hypothyroidism was not associated with cardiovascular events or all-cause mortality, which indicated that thyroid disorders had no influence on the risks of myocardial infarction and stroke. This study led researchers to conclude, ‘Regularly assessing a patient’s thyroid status may help estimate the cardiac risk of dialysis patients.’
